# Diagnosis of DVT in the emergency department: time spent on diagnosis

**DOI:** 10.1186/1757-7241-20-S2-P13

**Published:** 2012-04-16

**Authors:** Christian Skjærbæk

**Affiliations:** 1 Department of Medicine, Regionshospitalet Viborg, Denmark

## Background

Electronic white-boards could be important tools in the monitoring of patient-flow in the emergency department. Ideally data from the electronic white-boards could be used to describe the course of events for different groups of patients, allowing us to identify “bottlenecks” and possible optimise workflows. The aim of this study was to describe the time course in the emergency department for patients with suspected deep venous thrombosis (DVT) who at the same time were suitable for out-patient management.

## Methods

All patients admitted to our department with suspected DVT in 2010 were identified from our electronic white-board, delivered by Logica, Denmark. Patients who were discharged directly from the emergency department and who did not receive any treatment that could only be done in-hospital such as intravenous therapy were considered suitable for out-patient management. From the electronic whiteboard data were obtained for the time of the following events: admission, the patient being seen by a doctor, ordering of blood testing, return of answer of blood testing, ordering of diagnostic imaging, and discharge. Data are given as median and 25th and 75th percentile.

## Results

We identified 252 patients with suspected DVT and 192 (76%) were considered suitable for out-patients management. Median time from admission to the patient first being seen by a doctor was 54 (25-111) minutes. Time from admission to blood testing was ordered was 25 (14-35) minutes and time to answer was 80 (55-112) minutes. Time from admission to discharge was 21.5 (8.7-25.8) hours for patients who had ultrasound examination and 5.0 (3.8-11.0) hours for patients without ultrasound examination (p<0.05).

## Conclusion

The use of data from the electronic white-board to describe the time course of diagnostic work-up in the emergency department was successful. The total time used for diagnosis of suspected DVT was increased more than fourfold if ultrasound examination had to be done. Waiting for the patient to be seen by a doctor or waiting for the results of blood testing had little influence on the time used. A model for out-patient management of patients suspected of DVT will be presented and could possibly reduce the total time spent on the diagnosis.

